# A new measure based on degree distribution that links information theory and network graph analysis

**DOI:** 10.1186/2042-1001-2-7

**Published:** 2012-06-24

**Authors:** Michael W Hadley, Matt F McGranaghan, Aaron Willey, Chun Wai Liew, Elaine R Reynolds

**Affiliations:** 1 Neuroscience Program, Lafayette College, Easton, PA 18042, USA; 2 Department of Computer Science, Lafayette College, Easton, PA 18042, USA

**Keywords:** Degree distribution, Graph theory, Information integration theory, Neural networks, Degree distribution, Small world properties

## Abstract

**Background:**

Detailed connection maps of human and nonhuman brains are being generated with new technologies, and graph metrics have been instrumental in understanding the general organizational features of these structures. Neural networks appear to have small world properties: they have clustered regions, while maintaining integrative features such as short average pathlengths.

**Results:**

We captured the structural characteristics of clustered networks with short average pathlengths through our own variable, System Difference (SD), which is computationally simple and calculable for larger graph systems. SD is a Jaccardian measure generated by averaging all of the differences in the connection patterns between any two nodes of a system. We calculated SD over large random samples of matrices and found that high SD matrices have a low average pathlength and a larger number of clustered structures. SD is a measure of degree distribution with high SD matrices maximizing entropic properties. Phi (Φ), an information theory metric that assesses a system’s capacity to integrate information, correlated well with SD - with SD explaining over 90% of the variance in systems above 11 nodes (tested for 4 to 13 nodes). However, newer versions of Φ do not correlate well with the SD metric.

**Conclusions:**

The new network measure, SD, provides a link between high entropic structures and degree distributions as related to small world properties.

## Background

The nervous system is an informational system on the grandest scale: complex both in terms of its number of components and its organization. To understand how information is processed within it, physiologists and modelers have traditionally examined the electrical dynamics that directly convey information across the components of the system (that is, firing patterns of neurons or groups of neurons). With advancements in imaging technology, more recent work has focused on the structural properties of networks of neurons (the physical connections between neurons) that underlie these functional dynamics. Several efforts are underway to understand both structural and functional connections of the brain and how that connectivity influences informational flow and capacity. A group of researchers, now collectively part of the Human Connectome Project, has been creating connectivity maps of model and human nervous systems and developing tools to analyze their informational, structural and functional properties [1-5].

The graph theory metrics used in these analyses are based on the general properties of complex networks (that is, nonrandom, nonlattice networks) [6]. Some measures, such as degree, quantify the number of connections or edges between nodes. More complex measures look at patterns of connections: whether all the nodes of the system are closely connected or integrated (for example, path length - the minimum number of edges between nodes); or whether some parts of the graph might have clustered connections or hubs (for example, clustering coefficient - a measure of how related neighbors are in a graph). These metrics not only provide a way of analyzing networks but also lay the groundwork for understanding them. Based on these models, metrics, and physical data of connectivity in the brain, it has been proposed that neurological networks have small world properties, that is they are a collection of interconnected hubs [7-10]. Small world characteristics are found in many real world networks [11].

A different approach to understanding structure-function relationships of neural networks is to use information theory to provide a theoretical framework for identifying structures that integrate information while allowing for the differentiation necessary for a highly complex information capacity [12]. Tononi and collaborators created a metric called Phi (Φ), a measure of the minimum effective information (EI). EI is a directional measure of the causal influences between subsets of a network, and thus the minimum EI reflects a system’s capacity to integrate information [13,14]. The Φ measure, which was updated in 2008 and in 2011, moved the integrated information theory closer to the goal of formalizing a theory of consciousness based on the ability of a system to integrate and process large amounts of information [15-17]. Graphs that have been optimized for high Φ have been proposed to have small world properties suggesting that the structures found within biological neural networks would also have the ability to integrate information. However, this finding needs to be confirmed with the newer derivations of Φ [13]. Much of the literature in this area of complexity and consciousness focuses on a neural network having properties of integration and differentiation that would be apparent at both the structural and functional levels of analysis [18].

It seems then that some common structural characteristics may underlie complex neural networks: the ability of each node to reach any other node (integration or connectedness), and a high degree of node structure variance (specialization or differentiation). Currently there is no direct measure of these properties. The small world properties of a system of nodes are calculated from the ratio of the graph metrics for clustering (a measure of intermodal connectivity) and pathlength (a measure of the average distance between nodes). The ratio is normalized with corresponding values from a ‘random’ system [11]. This measure for small worldness may or may not reflect the properties of connectedness and specialization seen within real world systems. The goal of our work was to capture the characteristics of connectedness and segregation in a new variable that can be used to bridge the graph theory measures and the information theory metrics for larger nodal systems. Our new metric, System Difference (SD), is a Jaccardian measure of difference across a system that reflects the degree distribution of a network. We discuss this new measure in terms of its mathematical properties and its predictive value for structural properties based on other graph metrics, and we compare our new measure to other measures of complexity. When analyzing a population of randomly generated nodes, high SD is predictive of structures that are connected, but maintain some clustering, much like those structures that have small-world properties.

## Results and discussion

### Development of new variables

We considered a number of options for generating variables that were computationally simple yet captured the properties of specialization and connectedness. Information theory, which uses entropy as a basic means to understand the information flow within a system, was first developed by Shannon in 1948 [19]. The application of this theory to networks formalizes that idea that a system’s entropy quantifies the number of possible states available to the system. So we were looking for a metric that measured specialization and connectedness, but also took into account that systems with an intermediate density of connections were likely to be the most complex in terms of information states. We generated random networks and examined the graph properties of various metrics. We tested density, variables based on cycles, number of reciprocal connections, and several modularity measures derived from the igraph and other sources [18,19]. Two newly defined measures best captured the features of connectedness and specialization: Average Connectedness (AC) and SD.

**Figure 1:**
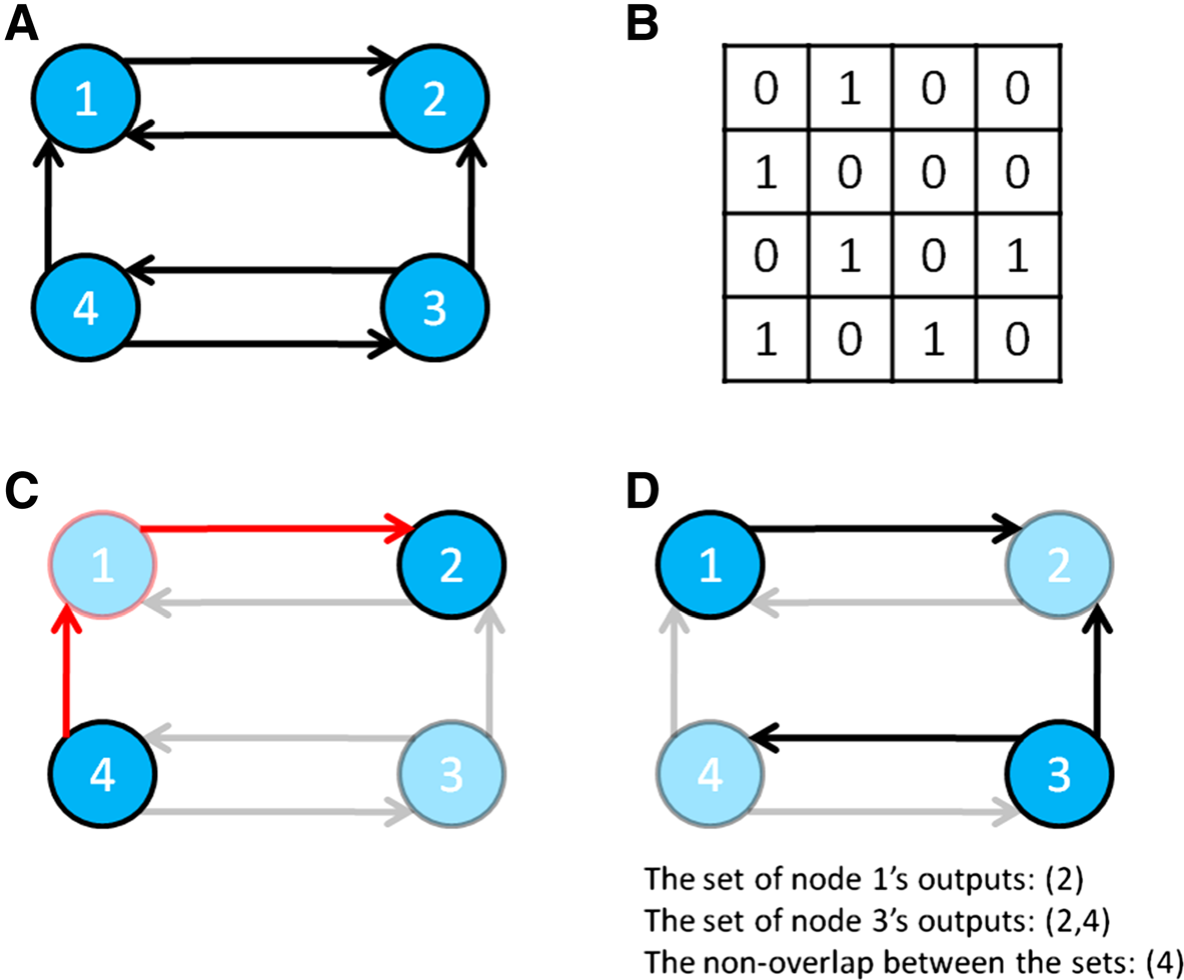
**Calculations of System Difference and Average Connectedness in a graph. A)** Representation of a directed, binary system in graph form.** (B)** The same system's corresponding connection matrix where rows represent outputs and columns represent inputs. A nonzero value in **A**_*i,j*_ indicates the presence of a connection from node *i* to node *j*, and a value of zero indicates no connection. **(C)** Illustration of the concept of reachability. A node is reachable from another node if a path can be found between them. Node 4 can reach node 2 by passing through node 1. Node 2 cannot reach node 4. Average Connectedness is the average node reachability of the graph (that is, the number of pairs of nodes A and B - such that B is reachable from A - divided by the number of nodes). **(D)** The central calculation of System Difference (SD): non-overlap. When comparing the output structure of node 1 to that of node 3, we must define sets representing each node’s outputs. Those sets can then be compared to find the number of different entries (or non-overlap) between them. SD is the average non-overlap for all combinations of two nodes in the graph.

In analyzing networks, we can represent the network as a graph with circles representing nodes (or neurons in the case of the nervous system) and arrows representing directed edges or connections (Figure 1A). It is easier to work computationally with a network system represented as a matrix, **A**, where a nonzero entry indicates a connection and where the columns represent inputs and the rows represent outputs (Figure 1B). Our variable capturing connectedness, (AC), was defined as the ability of a node to communicate information, either directly or through a series of nodes, to any other node in the network (See Figure 1C for a sample calculation). AC is the average reachability of a system. To implement this computationally, we created a matrix, **R**, of reachabilities (using the transitive definition) where a zero in position **R**_*i,j*_ indicates that node *j* is not reachable from node *i* and a one in position **R**_*i,j*_ indicates that node *j* is reachable from node *i*. AC is the sum of the elements in the matrix divided by n^2^[20].

Our variable capturing specialization, (SD), quantifies the average difference in connection patterns between any two nodes of a network (see Figure 1D for a sample calculation). We represent each node’s inputs and outputs as sets and apply a variant of Jaccardian distance to count the non-overlap in the input and output sets between pairs of nodes. SD is the average non-overlap in input structure plus the average non-overlap in output structure.

Comparisons were made between the new measures, AC and SD, using a small, randomly generated sample of weakly connected, directed matrices of 4 to 13 nodes. In generating these matrices, the probability of a connection was 0.5, and there were no self-loops (**A**_*i,i*_ = 0). Isomorphs and non-weakly connected systems were excluded (see Methods). A weakly connected, directed graph is a graph in which each node can reach all other nodes if you were to treat directed connections as bidirectional or undirected. Based on regression analysis at seven nodes, AC and SD were highly correlated with each other - AC accounted for over 80% of the variance in SD. Given the correlation between these variables, we continued forward with the SD metric.

SD was formulated to measure specialization in particular by quantifying the differences in connection structure between the nodes in an unweighted, directed graph. One might expect that two neurons that perform similar functions are more likely to be connected to similar sets of neurons and likewise, two neurons performing divergent functions are less likely to be connected to similar sets of neurons. A simple way to capture this dissimilarity of sets is through Jaccard Distance. Jaccard Distance (shown in Equation 1) is the percentage of distinct elements that are not shared between two sets [21]:

(1) $$\begin{array}{c} A\;and\;B\;are\;set\;and\;\left|X\right|\;is\;the\;size\;the\;set\;X\\ JaccardDistance\left(\text{A,B}\right)=1-\frac{\left|A\cap B\right|}{\left|A\cup B\right|}\\ \;=\frac{\left|A\cup B\right|-\left|A\cap B\right|}{\left|A\cup B\right|} \end{array}$$

The numerator of the Jaccard Distance from Equation 2 calculates the number of distinct elements (the non-overlap) in the sets A and B:

(2) $$Nonoverlap\left(\text{A,B}\right)=\left|A\cap B\right|-\left|A\cup B\right|$$

Since SD is a measure of the average non-overlap between nodes in a system, it can be derived through repeated application of Equation 2 to sets that represent the connections in a graph. These sets can be quantified as:

$$\begin{array}{c} {\text{Out}}_{x}\text{is}\;\text{the}\;\text{set}\;\text{of}\;\text{nodes}\;\text{that}\;\text{node}\;x\;\text{projects}\;\text{to}\\ {\text{In}}_{x}\text{is}\;\text{the}\;\text{set}\;\text{of}\;\text{nodes}\;\text{that}\;\text{project}\;\text{to}\;\text{node}\;x \end{array}$$

SD is the average difference (or non-overlap) in the sets and can be formalized as:

(3) $$\text{SD}=\frac{\text{non}-\text{overlap}\;\text{of}\;\text{outputs}+\text{non}-\text{overlap}\;\text{of}\;\text{inputs}}{\text{number}\;\text{of}\;\text{comparisons}}$$

The non-overlap of the sets representing outputs and inputs is obtained by summing the non-overlaps over all distinct pairs of nodes in the graph. In order to obtain an average difference, the number of non-overlap must be divided by the number of distinct comparisons. There are n/2 comparisons where *n* is the number of nodes which can be simplified to the denominator in Equation 4. By substituting Equation 2 into the numerator of Equation 3, the final SD formula becomes:

(4) $$\text{SD}=\frac{\left(\sum_{a=1}^{n}\sum_{b=a+1}^{n}non-overlap\left(Out_{a},Out_{b}\right)\right)+\left(\sum_{a=1}^{n}\sum_{b=a+1}^{n}non-overlap\left(In_{a},In_{b}\right)\right)}{\frac{n\times \left(n-1\right)}{2}}$$

One advantage of SD is that it is computationally simple. We calculated the computation times for SD with increasing number of nodes from benchmark experiments. SD can be calculated for a 15 node matrix in 0.0001 seconds and for a 1000 node matrix in under a minute. The computationally fast measure SD can be used to assess larger networks and therefore may be a good measure for assessing biologically based networks and other complex nodal systems.

### Comparisons of System Difference with graph theory metrics

**Figure 2:**
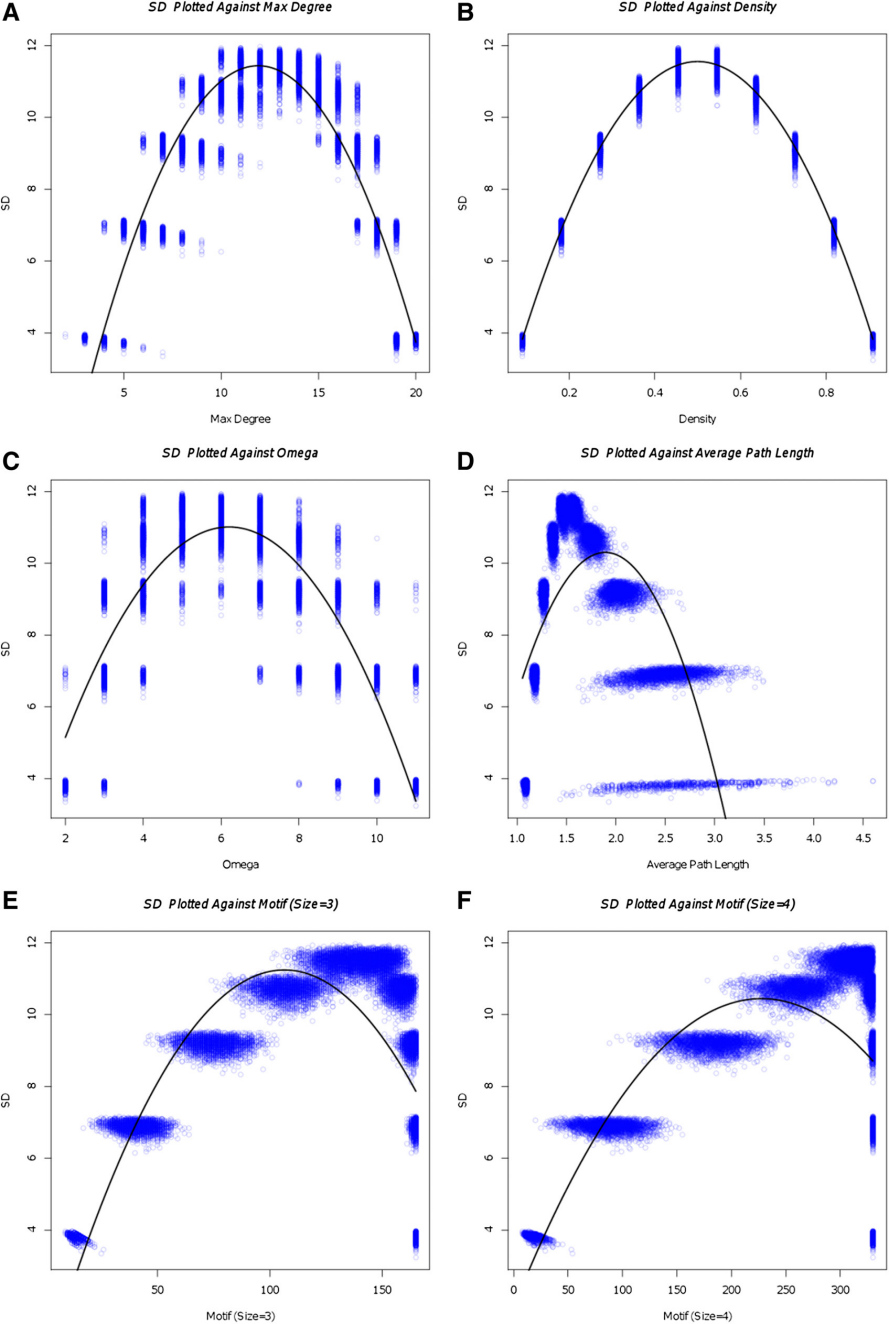
**SD Plotted Against Graph Metrics.** Each graph is generated by calculating System Difference and a graph metric in 24,000 random, weakly connected, normalized networks (n = 11). We sampled from graphs with degrees ranging from 1 to n-1, in steps of one such that the normalized graphs would only have one weight value (see Methods: System Difference comparisons with graph theory metrics (Figure 2) and 2003 Phi. Comparisons with Graph Variables'). The lines plotted on the graphs are the best fits for the second-degree polynomials of SD. SD is plotted against **A)** maximum degree, **B)** density, **C)** omega, **D)** average path length, **E)** motif number (size three) and **F**) motif number (size four).

Using a large sample of random, directed networks (24,000 networks with 8 to 11 nodes), we looked at the relationship of SD to a number of graph theory measures. Each matrix, **A**, (of size n) was generated by creating n columns each randomly filled with c connections where c is a random number from 1 to n-1 inclusive (c was the same for each matrix). Self loops were eliminated by inserting zero on the diagonal. This generation procedure produces matrices that are both normalized and binary (see Methods for further explanation). We used the igraph software package to generate the values of the graph metrics for the sample graphs [22]. SD was plotted against the graph theory measures for density, maximum degree, omega, number of motifs (of sizes three and four), and average path length (each of these measures are defined and discussed below). The relationships between SD and each graph metric for 11 nodes were fitted with a polynomial regression, and the second-degree fits are presented along with the individual data points in Figure 2.

Density and degree are fundamental properties of a system; density is the percentage of connections present in a graph, and degree is the number of edges connected to a node. In Figure 2A and B, we graphed SD against the density and maximum degree of the graph (the maximum of the sum of in- and out-degrees over all nodes). For density and degree, SD shows an upside-down U-shaped relationship. This trend suggests that SD shows entropic properties over degree distribution (high and low degrees yield low SD while median degrees yield high SD). As described above, we would expect that a graph with no connections (all nodes are independent) and a fully connected graph (excessive information communication causing every node to carry the same information) would have less complexity than matrices with an intermediate number of connections (a balance of information communication and distribution of information).

A clique is a subset of nodes in a graph that are fully connected if you replace all the directed edges with bidirectional edges. A maximal clique is a clique that is not contained within a larger clique. In order to get a sense of modularity, we plotted SD against omega (the size of the largest maximal clique) as shown in Figure 2C[22,23]. Specialization and connectedness are reflected in a balance between local, modular structures and more global structures. SD appears to capture this trend -showing omegas of approximately six at maximal SD indicating some modular structure.

Average path length is the average geodesic distance between two nodes in a graph (that is, the average number of edges that must be traversed to travel from one node to another) [22]. Looking at Figure 2D, we can see that SD shows a skewed upside-down U-shaped distribution against average path length. High SD appears to be biased towards low average path length. A low average path length indicates that the system has strong global connections and so the distribution of values for average pathlength suggests that SD might be an integrative measure. Hub or modular structures with shorter pathlengths are characteristics of small-world networks typical of biological systems [9].

Lastly, we looked at the presence of certain structural motif metrics. A structural motif is a weakly connected, directed graph of n nodes that serves as a building block within a larger graph. Weak connectedness requires that a motif have at least n-1 connections, and since self-loops are excluded, a motif can have at most n^2^-n connections. For each size n, there are a fixed number of unique (non-isomorphic) motif classes. A graph can be analyzed to count the number of times each of the motif classes (of size n) occurs; this metric is called motif number [24,25]. In Figure 2E and F, we plotted SD against motif number for motifs of size three and size four respectively. High SD is correlated with higher number of motifs (the curve shifts to the right), which suggests that SD might be indicative of clustering or hub structures within the larger system.

The data presented here suggest that high SD networks are biased towards shorter pathlengths and higher clustering, structural properties associated with small world properties.

### System Difference and degree

In all the analyses of SD, we observed a strong relationship with the density or degree of the graph. The U-shaped trend that SD shows against density becomes more pronounced as the number of nodes is increased. In exploring SD’s relationship to degree, we discovered that SD could be expressed in terms of degree distribution.

A graph can be represented with a connectivity matrix - a matrix **A** where A_ij_ represents the connection from node *i* to node *j*. In our unweighted graphs, A_ij_ will have a value of one if node *i* has an output to node *j* or A_ij_ will have a value zero if no such output exists. The set theory formulation of SD (Equation 4) can be represented as pairwise comparisons within the connection matrix where:

$$A\;\text{is}\;\text{the}\;\text{connection}\;\text{matrix}\;\text{and}\;D\left(x,y\right)=\{\begin{array}{l} 1\;if\;x\neq y\\ 0\;if\;x=y \end{array}$$

The difference function *D*(*x*, *y*) simply compares *x* and *y* and returns a one if *x* and *y* have the same value and a zero otherwise. The non-overlap functions from the numerator of Equation 4 can be substituted with the difference function. The non-overlap in outputs between nodes *a* and *b* can be calculated by comparing row *a* and row *b* of **A**, and the non-overlap in inputs can be calculated by comparing column *a* and column *b*:

(5) $$\text{non}-\text{overlap}\left({\text{out}}_{a},{\text{out}}_{b}\right)=\sum_{t=1}^{n}D\left(A_{a,t},A_{b,t}\right)$$

(6) $$\text{non}-\text{overlap}\left({\text{In}}_{a},{\text{In}}_{b}\right)=\sum_{t=1}^{n}A_{a,t},A_{t,b}$$

This substitution can be used in Equation 4 to arrive at a derivation of SD in terms of **A**:

(7) $$SD=\frac{\left(\sum_{a=1}^{n}\sum_{b=a+1}^{n}\sum_{t=1}^{n}D\left(A_{a,t},A_{b,t}\right)\right)+\left(\sum_{a=1}^{n}\sum_{b=a+1}^{n}\sum_{t=1}^{n}D\left(A_{t,a},A_{t,b}\right)\right)}{\frac{n\times \left(n-1\right)}{2}}$$

The order of summation in the numerator of Equation 7 can be rearranged to show that the non-overlap of the outputs is a computation that can be performed for each column independently:

(8) $$\sum_{a=1}^{n}\sum_{b=a+1}^{n}\sum_{t=1}^{n}D(A_{a,t}A_{b,t})=\sum_{t=1}^{n}\sum_{a=1}^{n}\sum_{b=a+1}^{n}D(A_{a,t}A_{b,t})$$

For any given *t*, the right hand side of Equation 8 represents the non-overlap in column t, which amounts to the number of times that **A**_*a,t*_ and **A**_*b,t*_ are not equal for each combination of *a* and *b*. This can be found by multiplying the number of inputs in the column by the number of locations where there is no input. The number of inputs in a column *t* is the in-degree of node *t*:

(9) $$\text{in}-\text{degree}(t)=\sum_{x=1}^{n}A_{x,t}$$

The number of locations where there is no input can be found by subtracting the in-degree from *n* (the number of nodes). Then the non-overlap in column *t* becomes:

(10) $$\sum_{a=1}^{n}\sum_{b=a+1}^{n}D(A_{a,t},A_{b,t})=\text{in}-\text{degree}(t)*(n-\text{in}-\text{degree}(t))$$

A similar rearrangement shows that non-overlap of the inputs can be found from each row independently. The end formula is the same except with out-degree used instead of in-degree, where out-degree is:

(11) $$\text{out}-\text{degree}(t)=\sum_{x=1}^{n}A_{t,x}$$

With substitutions for both the non-overlap in the inputs and in the outputs, the SD calculation can be expressed completely in terms of degree:

(12) $$SD=\frac{\sum_{t=1}^{n}\left(\text{in}-\text{degree}(t)\times \left(n-\text{in}-\text{degree}(t)\right)+\text{out}-\text{degree}(t)\times \left(n-\text{out}-\text{degree}(t)\right)\right)}{\frac{n\times (n-1)}{2}}$$

### System Difference and graph substructure

**Figure 3:**
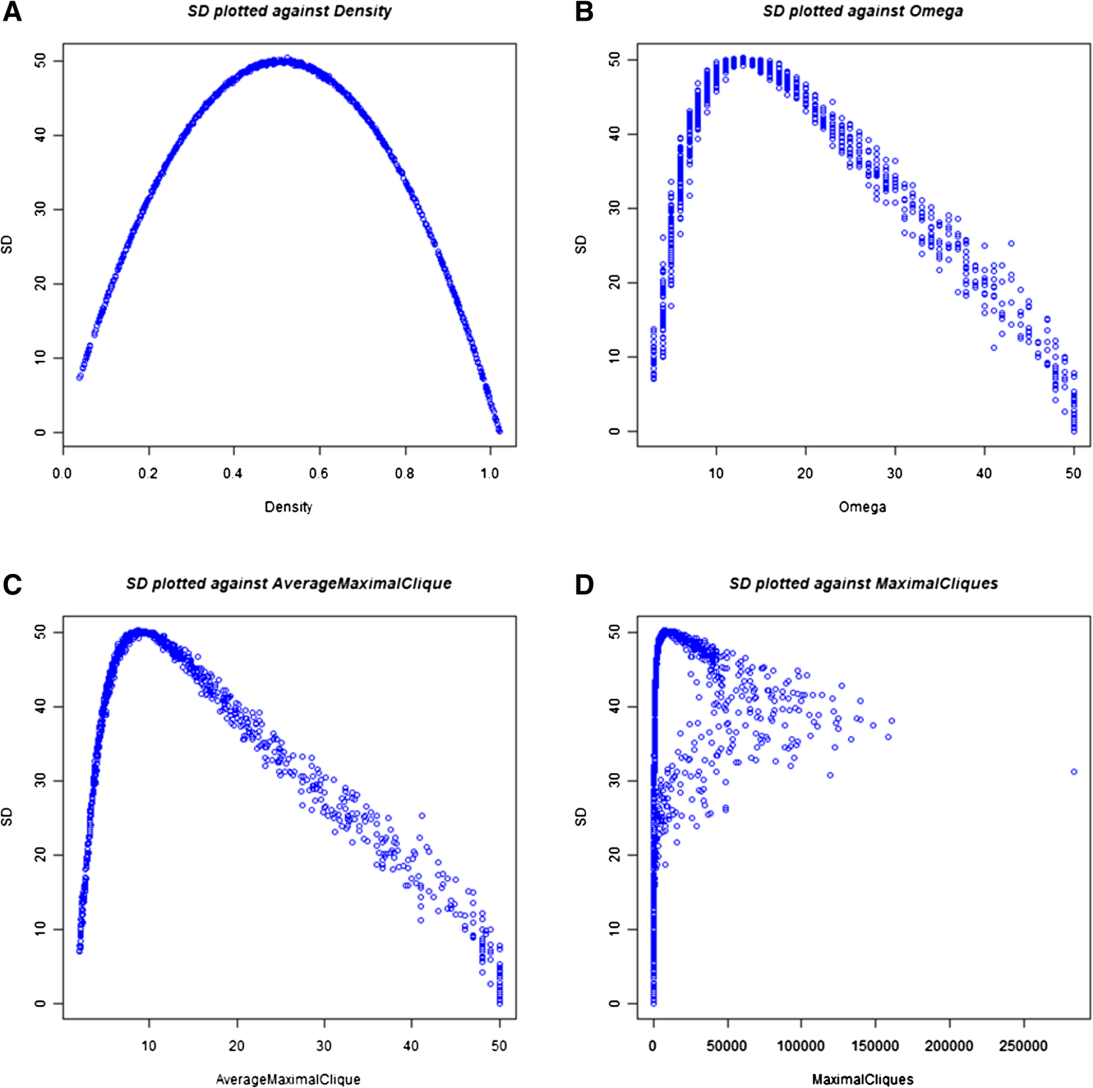
**System Diffence plotted against density and clique graph metrics.** Each graph is generated by calculating System Difference (SD) and a graph metric in 1000 random, weakly connected networks (n = 50) sampled uniformly over density. A random density value was generated for each matrix by adding connections one at a time until the random density was reached or just exceeded. ** (A)** SD plotted against density.** (B)** SD plotted against omega. **(C)** SD plotted against average maximal clique.** (D)** SD plotted against number of maximal cliques.

Our understanding of SD in terms of degree suggests that the substructure of a graph should show certain trends with respect to SD. Recall that cliques are subsets of nodes that are fully connected in an undirected version of the graph, a maximal clique is a clique that is not contained within a larger clique and omega is the size of the largest maximal clique within a graph. For these experiments, a new set of random, directed, and weakly connected matrices were generated that sampled uniformly over density. Random density was generated for each matrix by adding connections one at a time until the random density was reached or just exceeded. Again, isomorphs, self-loops and non-weakly connected systems were excluded (see Methods). When SD, with a graph size of 50, is plotted against omega (or average maximal clique size) the trend is U-shaped; thus, structures with low SD either have a very small omega or very large omega (Figure 3B, C). When SD is plotted against the number of maximal cliques, the trend is more complicated (Figure 3D). Larger numbers of maximal clusters bias networks to higher values of SD, but low numbers of maximal cliques span the whole range of SD values. Structures with maximal SD have clique sizes of about 10 to 15. This means that in these high SD structures there are groups of at least 10 nodes that are interconnected. In the lowest SD structures, either all the nodes form one interconnected group or none of the nodes form a connected subset. The combined results from the individual trends in Figure 3B, C, D suggest that high SD networks tend to have multiple large cliques suggestive of a modular structure.

**Figure 4:**
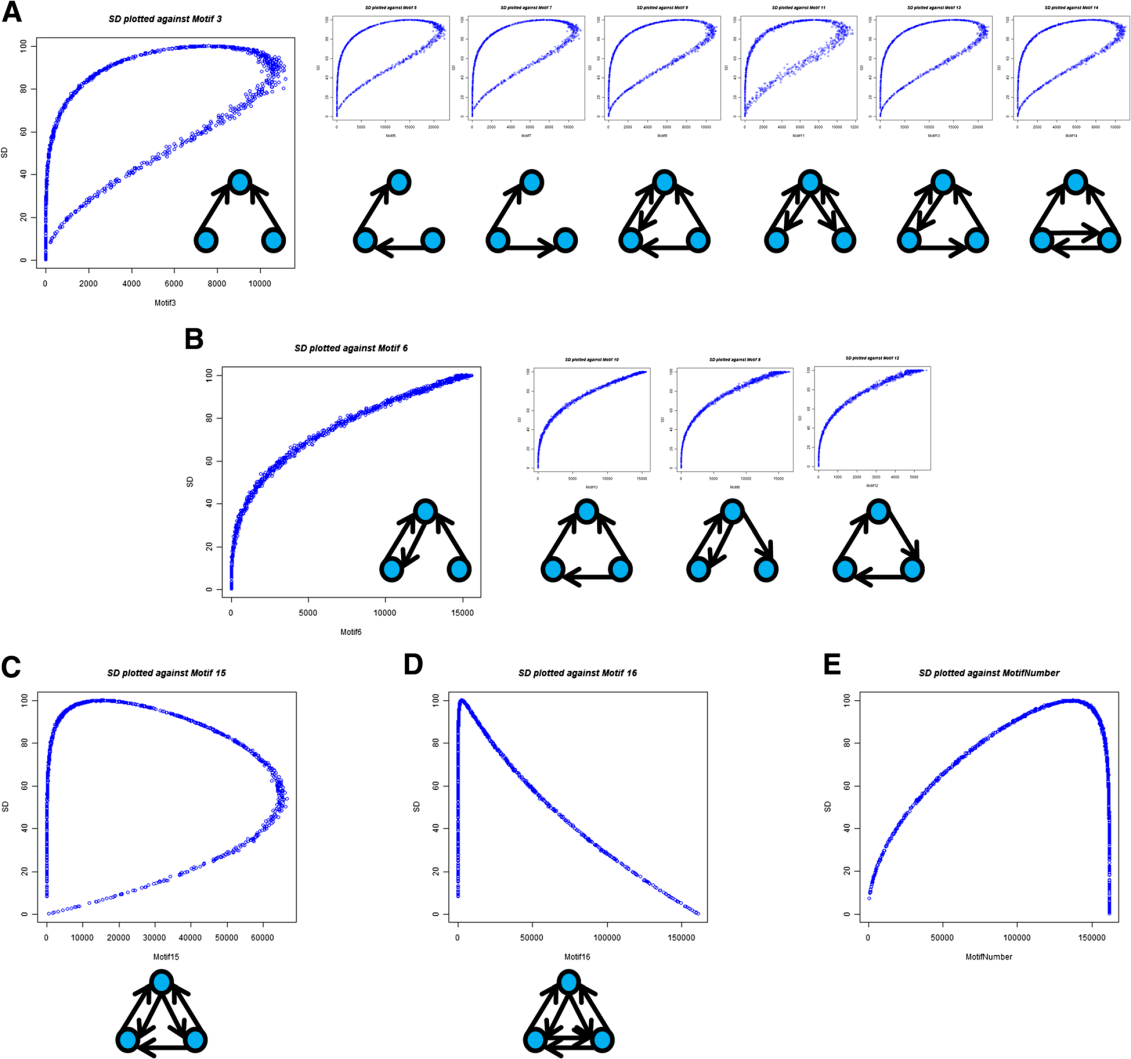
**System Difference plotted against motifs.** Each graph is generated by calculating System Difference (SD) and metric in 1,000 random, weakly connected networks (n = 100) sampled uniformly over density. A random density value was generated for each matrix by adding conncetions one at a time until the random density was reached or just exceeded. (**A-D**) SD plotted against the number of occurences of different motifs of size three. The number convention comes from igraph [22], so network graphs are displayed for clarity. The plots and network graphs are grouped by the trend observed against SD. (**E**) SD plotted against the motif number for motifs of size three.

A closer analysis of SD and motifs also gives us a better understanding of graph substructure. Recall that motifs are structural subunits that can be identified within a graph. The distribution of SD plotted against different motif metrics emphasizes that SD is a measure of degree distribution. Four distinct trends among the 13 different motifs classes are observed when comparing the frequency of the motif structures (of size three) to SD, each of which is related to the density of that group. Group 1 (Figure 4A) contains all motif structures that have either two or four connections (densities of 1/3 or 2/3 respectively, which equally deviate from a density of 0.5). Group 2 (Figure 4B) contains all motif structures that have three connections (density of 0.5). Group 3 (Figure 4C) has the motif with five connections (density of 5/6). Finally, group 4 (Figure 4D) has the motif with six connections (density of 1). High SD structures are saturated in groups 1 and 2 while generally lacking in groups 3 and 4. Low SD structures are generally lacking in groups 1 and 2 and either saturated or deficient in groups 3 and 4.

The trend that motifs show is that a density of about 0.5 maximizes SD on a local, motif scale. The entropic properties of SD yield the symmetrical properties observed when comparing over-connected and under-connected structures. The local emphasis on density in specific substructures then results in the motif number trend observed (Figure 4E), which is a slightly skewed version of the trend that overall density shows against SD (Figure 3A). These results suggest that maximum SD is obtained when the in-degree and out-degree for each node are as close to n/2 as possible, and minimum SD is obtained when the degrees are as far from n/2as.

The analysis of SD in terms of substructure can be taken to the extreme of examining the contribution by individual nodes. If we temporarily assume that in-degree and out-degree for each node can be set independently from any other node, we can take the analysis of SD in substructure to the extreme of a single node:

(13) $$\begin{array}{c} SD\;\text{of}\;{\text{node}}_{t}=\text{in}-\text{degree}(t)\\ \;\times \left(n-\text{in}-\text{degree}(t)\right)\\ \;+\text{out}-\text{degree}(t)\\ \;\times \left(n-\text{out}-\text{degree}(t)\right) \end{array}$$

These results lead to a general strategy for hitting a target SD by either moving in-degrees and/or out-degrees away from or towards n/2. This strategy leads to the insights of the nuances of the global trend of SD and density. Random graphs with a density of 0.5 will likely have a high SD value because in- and out-degree will tend towards n/2. In contrast, a graph with a density near 0.5, but with in-degree and out-degree distributions that differ greatly from n/2 will actually yield a low SD.

**Figure 5:**
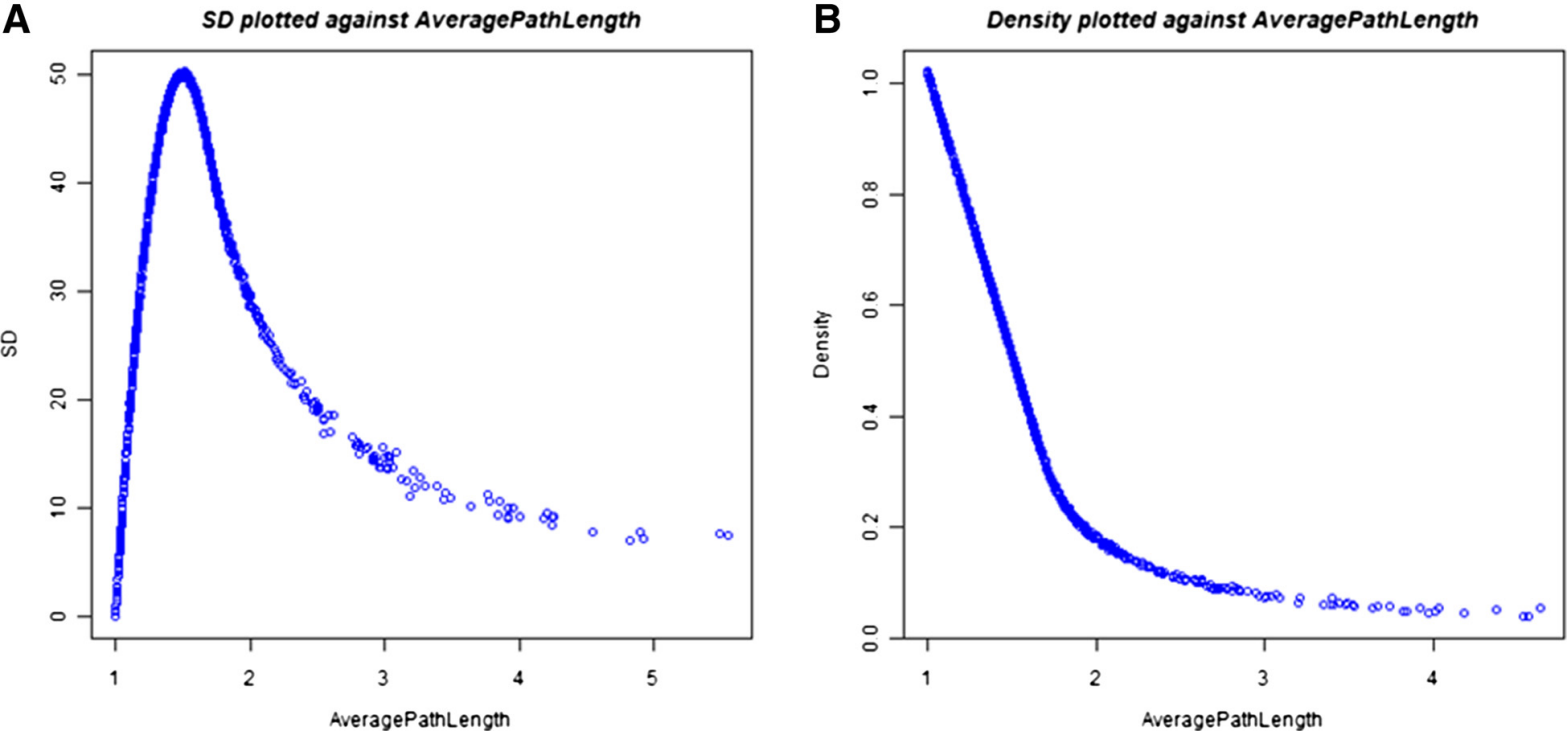
**System Difference, density and path length.** The graphs are generated by calculating System Difference, density and average path length in 1,000 random, weakly connected networks (n = 50) sampled uniformly over density. A random density value was generated for each matrix by adding connections one at a time until the random density was reached or just exceeded. **(A)** SD plotted against average path length.** (B)** Density plotted against average path length.

In terms of the integrative measure of average path length, SD generally follows a skewed upside-down U-shaped trend with the peak at a path length of 1.5 (Figure 5A). The graph shows a long tail as path length increases because graphs are required to be weakly connected. The section of the curve (to the left side of the peak) represents the matrices with densities of 1 to around 0.5. Networks that are fully connected or near fully connected have path lengths near 1, that is, every node is directly linked to nearly every other node.

Density and average path length have a relationship with a critical point (Figure 5B). With densities of 0 to 0.3, adding additional edges results in large, decreasing gains in average path length. From 0.3 to 1, adding edges results in much smaller (but constant) decreases in path length. The critical point is around 0.3 density (which corresponds to a path length of around 1.8) when the critical gaps in network connectivity have all been filled in. Although the maximal SD lands off this critical point, structures around this critical point have high SD, representing a balance between density and path length.

The trends seen between SD and substructure/average path length can be combined. The average path length for the nodes within a clique should approach one since the nodes are interconnected. Since high SD matrices with 50 nodes have maximal cliques around 15, at the very least one third of the nodes are weakly connected, which would likely generously decrease the average path length. The path length would be lowered even more if there were multiple maximal cliques that were linked together.

### System Difference and other complexity measures

Our original inspiration in defining SD was the measure of complexity originally developed by Tononi and collaborators, Φ (here after referred to as 2003 Φ) [12,13]. 2003 Φ is a measure of the dynamic informational properties of a network; high 2003 Φ strikes a balance between the diversity of information states of a network and the causal dependence between the nodes of the network [13]. 2003 Φ is a measure of the integrative capacity of a set of nodes. The causal interactions are captured by partitioning the set into two subsets, and then the entropy of firing is measured in one subset while the other subset is stimulated with maximal entropy of activity. This information flow over a bipartition, the IE, is modified version of mutual information that takes into account the direction of information flow. The 2003 Φ for a subset of system, S, is defined as the bipartition of S such that EI is minimized. (See Methods2003 Phi.) [13,14].

Several recent papers, however, have called into question the calculation by which the 2003 Φ was derived. EI can be obtained from the covariance matrix of the network, which represents all deviations from independence among the nodes. In solving the linear equation representing the system dynamics, Tononi and colleagues made an assumption that was disputed first by Barnett *et al*. (2009) [26]. Various corrected versions of Φ have been offered most recently by Barrett and Seth in 2011 [16]. The extended version of Φ proposed by Barrett and Seth (referred to here after as Φ Empirical) calculates information based on an empirical, stationary distribution (see MethodsPhi Empirical). The approach of Barrett and Seth is based on taking the stationary firing of a system and calculating information integration of transitions from one state to another that is separated by some time lag, τ. Φ Empirical (given a particular τ) is the amount of information integration generated by the current state about the state τ time-steps in the past. It can be calculated either by observing a sufficient number of firing states or though an analytical formula [16].

We first assessed the relationship between SD and the 2003 Φ proposed by Tononi and collaborators. The matrices used were the same as those used to generate Figure 2 (24,000 random, directed networks with 8 to 11 nodes). In order to calculate 2003 Φ, all matrices must be normalized according the following equation (where **A** is the connectivity matrix before normalization and **C** is the normalized connectivity matrix):

(14) $$C_{\mathrm{ij}}=k\times \left(\frac{A_{\mathrm{ij}}}{\text{Sum}\;\text{of}\;\text{column}}\right)\text{where}\;k<1$$

As stated previously, each matrix **A** (of size n) was generated by creating n columns each randomly filled with c connections where c is a random number from 1 to n-1 inclusive (c was the same for each graph). We chose k to be 0.5, and thus each the connection weight was 0.5/c. This procedure enforces normalization (such that **C** = **A**) while keeping each matrix to only one weight value (in addition to the absence of a weight, that is, zero). SD is calculated by treating the matrix as if it is unweighted and hence, normalization does not change the value of SD. After generating these matrices and the values associated with them, we linearly regressed SD against 2003 Φ. The correlation values for the comparisons were 0.667 (n = 8), 0.804 (n = 9), 0.871 (n = 10), and 0.9140 (n = 11). Using various procedures for generating random networks, we consistently find the same level of correlation and the same trend that correlation increases with increasing number of nodes (data not shown). While the pattern of correlation is consistently strong across multiple experiments, the correlation is based on the general trends of 2003 Φ and SD rather than a point-by-point correspondence. Using the same set of matrices described above, we compared the 2003 Φ with the same graph metrics used in our analysis of SD. The 2003 Φ and SD appear to measure similar structural features of networks (trends in graphs of 2003 Φ plotted against the same graph metrics in Figure 2 closely match the trends SD shows in that figure), with one of the most notable shared features being a U-shaped distribution over density (data not shown).

**Figure 6:**
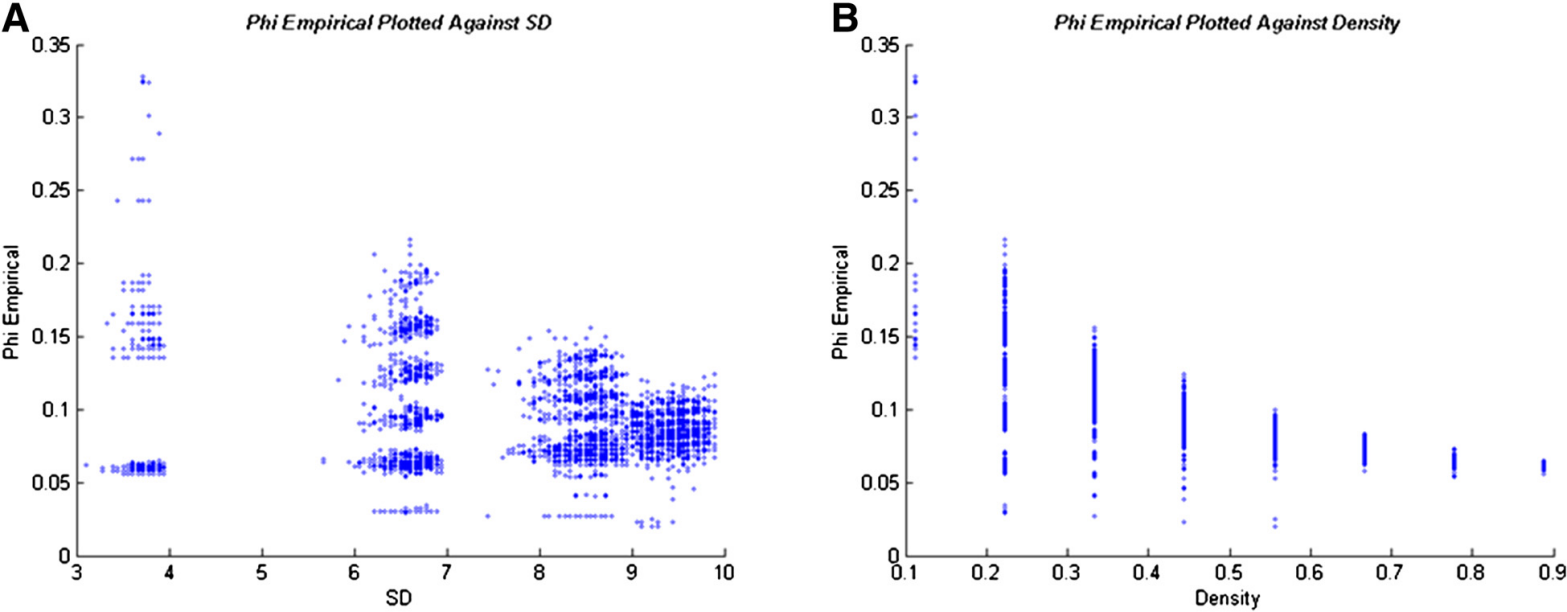
**Phi Empirical.** The graphs are generated by calculating Φ Empirical, System Difference and density in 5,000 random, weakly connected, normalized networks (n = 9) sampled uniformly over density such that the normalized graphs would only have one weight value (see Methods, System Difference and Phi Empirical).** (A)** Φ Empirical plotted against SD.** (B)** Φ Empirical plotted against density. Φ Empirical has a time lag parameter over which to calculate information integration. The results above are typical for all time lags attempted (one to four as well as the sum of the Φs over multiple time lags).

We also generated random matrices to compare SD, 2003 Φ and Φ Empirical using the same procedure for matrix generation and normalization described above that is, generating matrices such that **C** = **A**). We looked at correlations between these three measures and density. 2003 Φ and Φ Empirical differ in their trend over density. While 2003 Φ and SD share the entropic U-shaped distribution over density, Φ Empirical shows a less clear trend and does not correlate well with SD (Figure 6B). It appears that the highest Φ Empirical values are found in very low density networks. Consequentially, Φ Empirical does not correlate well with SD (Figure 6A). While it is clear that the calculation of 2003 Φ is flawed, the characteristics of Φ Empirical need to be further examined to assess whether it captures structural features associated with small world properties or the expectations associated with information theory.

## Conclusions

Degree distribution has always been recognized as an important aspect of structural characteristics of systems. Different defined types of systems (small world, scale free, modular) have distinctive degree distributions characteristics [3,27]. The neural complexity measure from which the original 2003 Φ was derived is heavily dependent on degree [28]. Many papers in the field define degree distributions as a probability function: the distribution is defined as the probability that a selected node has degree k [19,29]. The degree distribution of a system then represents the cumulative degree distribution as the probability over all nodes. The advantage SD offers over this method is that it specifically defines an entropic degree distribution where over-connected and under-connected distributions yield low SD and median connectivity results in high SD. We have not encountered a graph measure that links entropy and degree distribution.

SD then is a measure of entropic degree distribution and could be used to look for these specific features within graphs. It is possible and likely that a major determinant of neural complexity is degree distribution. It may be that a degree distribution that balances a set of constraints to produce a system that is both segregated and connected has a simple solution n/2, the optimal degree for complexity, as can be seen from our analysis of the graph measures in the context of optimum SD degree distribution. This combination of local and network degree optimums pushes local structures towards cliques, hubs or clusters with particular motif patterns and short path lengths. This makes sense from a biological perspective as well. We know from empirical evidence that the brain is modular and that it probably evolved as a series of subunits that were organized into the massively parallel system. Modules that met local degree optimum were hooked together in such a way as to create optimums at a higher level of the network hierarchy. So it makes sense that we would see this degree distribution at multiple levels of structure. As is so often in nature, the solution is elegant.

## Methods

### Matrix generation algorithms and experimental analysis

The generation algorithms for each experiment differed slightly. All experiments used directed, weakly connected graphs with no self-loops.

#### Development of the variables Average Connectedness and System Difference (no figure shown)

For each matrix that was generated, each connection had a 50% chance of being assigned a value of 0.5 and a 50% chance of being assigned a value of zero Self-loops, isomorphs and non-weakly connected systems (defined below in the section "Detection") were excluded. We collected datasets for networks of sizes 4 through 13 with sample size from 215 (n = 4) and 1,000 (n = 13). We performed exponential fits of AC on SD for each n using Microsoft Excel.

#### System Difference comparisons with graph theory metrics (Figure 2) and 2003 Phi

We generated a single set of matrices that could be used to make comparisons between SD, 2003 Φ and a set of graph theory matrices. In order to calculate Φ, matrices must be normalized. However, normalization does not affect the calculation of SD, which only takes into account whether nodes are connected or not. The matrices in these trials were generated according to our normalization procedure described in [13] and in more detail below. For each matrix that was generated, the number of weights per column was a random number between 1 and n-1 inclusive. n columns were created with n-1 elements (consisting of ones and zeros in random order). A main diagonal of zeros was inserted to form an n by n matrix with no self-loops. This procedure ensured that each matrix would only have one weight value (in addition to the absence of a weight, that is, zero). We collected datasets for networks of sizes 8 through 11 with a sample size of 24,000 for each n. For each network size, we calculated SD and 2003 Φ for each matrix as well as a selection of graph theory metrics (from igraph) [22]. We performed linear correlation of 2003 Φ on SD using the R statistics package [29]. We plotted SD and the graph theory metrics with second-degree polynomial model fits (Figure 2), and we also compared 2003 Φ to these same metrics (data not shown). Plots were generated in the R package [30]

#### System Difference time benchmarks

Randomly generated matrices for multiple node sizes were sampled. SD was calculated and the time to calculate was averaged over several sample sizes. These experiments were carried out on a set of identical rack mounted computer systems. Each system had two AMD Dual Core Opteron 275 processors with 8 GB of RAM.

#### System Difference and subgraph analysis (Figures 3, 4 and 5)

The matrices in these trials were generated to sample uniformly over density. A random density was generated for each matrix and connections were added one at a time until the random density was reached or exceeded. No normalization was applied. The graph theory metrics were calculated through igraph [22]. For motif metrics, 100-node networks were generated with a sample size of 1,000. For density, omega, average maximal clique size, number of maximal cliques and path length, 50-node networks were generated using a sample size of 1,000. Plots were generated in the R package [30].

#### System Difference and Phi Empirical (Figure 6)

The same matrix generation procedure was used as in the 2003 Φ comparisons with SD. In this case, 5000 nine-node systems were generated and then Φ Empirical, SD and density were calculated. Time lags of one ,two, three and four were used for Φ Empirical. We also calculated the sum of the Φ Empirical values over those time lags (see Φ Empirical below). The output was plotted using the R package [30].

### Detection

During generation of networks for any experiment, each newly generated network was checked against all previous networks generated in the experiment using the algorithms below. Networks that faiedl to pass the algorithms were discarded and a new random network was generated.

#### Isomorph detection

Two systems are isomorphic if a relabeling of the vertices of one system yields a copy of the other system. Isomorphs were avoided through igraph’s implementation of the VF2 algorithm for isomorph detection [22].

#### Weakly connected

A weakly connected, directed graph is a graph in which each node has an undirected path to each other node. Weak connectivity was checked using igraph’s connectivity detection implementation [22].

#### Self-loop

A loop (or self-loop) is a connection from a vertex onto itself. Loops were excluded by generating (n-1) by n matrices and then inserting a main diagonal of zero to form n by n matrices.

### Normalization

In calculating Φ, a normalization procedure was used to separate out the effects of weight magnitude from the effects of structure [13]. Their normalization takes a connection matrix **A**_,_ and generates **C**, the normalized connectivity matrix:

(17) $$C_{i,j}=k\times \left(\frac{A_{i,j}}{Sum\;of\;column_{j}}\right)\;where\;k<1$$

In order to preserve this normalization while keeping each matrix to only one weight value (in addition to the absence of a weight, that is, zero), we had to make the sum of the column *j* the same value for all *j*. By holding the number of weights per column, c, to a constant for all columns in a matrix, the resulting matrix is both normalized and binary. Since we chose k to be 0.5 (following the 2003 Φ protocol), each connection weight was 0.5/c. This procedure ensures that **C** = **A**. We applied the normalization procedure to all matrices in all experiments in which Φ was calculated.

### 2003 Phi

2003 Φ is a measure of the information capacity of a system based on the casual interactions within the system [13]. Φ($\hat{A}$) is the Φ of a subset, $\hat{S}$, of a stationary system, $\hat{X}$, whose connection matrix is specified by **CON(**$\hat{X}$**)**. A stationary system is one in which mean and variance firing do not change over time. The activity vector, $\hat{F}$, represents the activity of each of the elements of $\hat{X}$. The activity is governed by the following dynamics (when $\hat{R}$ is uncorrelated Gaussian noise with zero mean and unit variance and c is a constant):

$$\hat{F}=\hat{F}\;CON\left(\hat{X}\right)+c\hat{R}$$

The casual interactions of $\hat{S}$ are captured by EI, where EI for a bipartition of $\hat{S}$ into $\hat{A}$ and $\hat{B}$ is given by the following equations:

$$\begin{array}{l} EI\left(\hat{A}↔\hat{B}\right)=EI\left(\hat{A}\to \hat{B}\right)+EI\left(\hat{B}\to \hat{A}\right)\\ EI\left(\hat{A}\to \hat{B}\right)=MI\left({\hat{A}}^{H\max}:\hat{B}\right)\\ MI\left(\hat{A}:\hat{B}\right)=H\left(\hat{A}\right)+H\left(\hat{B}\right)-H\left(\hat{A},\hat{B}\right)\\ H\left(\hat{A}\right)=\frac{1}{2}\ln[{\left(2\times \pi \times e\right)}^{n}\det\left(\mathrm{cov}(\hat{A})\right)] \end{array}$$

where MI is mutual information, ${\hat{A}}^{H\max}$ is a system $\hat{A}$ where each element is substituted with independent noise sources of constrained maximum variance, H($\hat{A}$) is entropy of system $\hat{A}$, H($\hat{A}$,$\hat{B}$) is the joint entropy of systems $\hat{A}$ and $\hat{B}$, det(**C**) is the determinant of matrix **C**, and cov($\hat{A}$) is the covariance respectively of system $\hat{A}$. Φ($\hat{S}$) system in $\hat{A}$ and $\hat{B}$ such that the $EI\left(\hat{A}↔\hat{B}\right)$ is minimized:

$$\begin{array}{c} \text{MIB}(S)={\left[\hat{A};\;\hat{B}\right]}_{\hat{S}}\text{for}\;\text{which}\frac{EI(\hat{A}↔\hat{B})}{\min\{H^{\max}(\hat{A}),H^{\max}(\hat{B})\}}\\ \;=\min for\;all\;\hat{A}\;in\;\hat{S} \end{array}$$

where MIB is the minimum information bipartition, $\left[\hat{A};\;\hat{B}\right]$ is a bipartition of $\hat{S}$ into $\hat{A}$ and $\hat{B}$, $H^{\max}(\hat{A})$ is the maximum entropy available to $\hat{A}$, and min{…} is the minimum. Then the Φ for subset $\hat{S}$ is given as:

(21) $$2003\;\Phi (\hat{S})=EI\left(MIB\left(\hat{S}\right)\right)$$

For our analyses, we considered the subset of $\hat{X}$ with the greatest Φ to define the information capacity for $\hat{X}$.

Tononi *et al*. derived an analytical solution for finding the covariance of a system under stationary conditions [12]. Barnett *et al*. show the derivation to be erroneous (see [26] for a full description).

MATLAB code for the 2003 Φ (with the erroneous analytical solutions) is available from http://tononi.psychiatry.wisc.edu/informationintegration/toolbox.html[13]. We used the code as implemented with noise parameters c_p_ = 1 and c_i_ = 0.00001. These two values constitute the magnitude of noise from the first equation. When calculating $\text{EI}\left(\hat{A}\to \hat{B}\right)$, c_p_ is the magnitude of the perturbation noise applied to subset $\hat{A}$ while c_i_ is the magnitude of the intrinsic noise applied to subset $\hat{B}$. Putting this in the context of the first equation (given a system $\hat{X}$ bipartitioned into $\hat{A}$ and $\hat{B}$

(22) $$\hat{F}=\hat{F}CON\left(\hat{X}\right)+\hat{C}*\hat{R}$$

where ∗ is element-wise multiplication of vectors, and $\hat{C}$ is a column vector of size n (the number of elements in $\hat{X}$) such that:

(23) $${\hat{C}}_{j}=\{\begin{array}{l} c_{p}\text{if}\;\text{element}\;j\;\text{is}\;\text{contained}\;\text{in}\;\text{subset}\;\hat{A}\\ c_{i}\text{if}\;\text{element}\;j\;\text{is}\;\text{contained}\;\text{in}\;\text{subset}\;\hat{B} \end{array}$$

From the above equations describing the dynamics of the system $\hat{X}$, the 2003 Φ code uses an erroneous analytical solution to find EI. Thus the code takes noise parameters c_p_ and c_i_ as well as the connection matrix, **CON(**$\hat{X}$**)**, and returns a value for 2003 Φ. **CON(**$\hat{X}$**)** represents the normalized connection matrix which is alternatively noted in the body of our paper as **C**. **CON(**$\hat{X}$**)** (or **C**) is thus a matrix representing the strength of the weighted connections between elements of a system $\hat{X}$. In the context of 2003 Φ, these weights are applied to a system with linear dynamics.

### Phi Empirical

Given a general stationary Gaussian system, $\hat{X}$, the generative model is: ${\hat{X}}_{t}=A_{1}{\hat{X}}_{t-1}+A_{2}{\hat{X}}_{t-2}+\cdots +A_{p}{\hat{X}}_{t-p}+{\hat{E}}_{t}$

where **A**_*i*_ is the generalized connectivity matrix acting at different times and ${\hat{E}}_{t}$ is a stationary, Gaussian noise with zero mean and vanishing auto-covariance $\left(\mathrm{cov}\left({\hat{E}}_{t-\tau },{\hat{E}}_{t}\right)=0\;\text{when}\;\tau \neq 0\right)$. Much like the above 2003 Φ model, Φ Empirical is defined as the EI over the MIB. The EI with any time lag, τ, is given by the following equations (equations 0.33 and 0.34 from [16]):

$$\begin{array}{c} EI\left(\hat{X};\;\tau ,\left\{{\hat{M}}^{1},\;{\hat{M}}^{2}\right\}\right)=\frac{1}{2}\log\frac{\det\left(\mathrm{cov}\left(\hat{X}\right)\right)}{\det\left(\mathrm{cov}\left({\hat{X}}_{\text{t}-\tau }|{\hat{X}}_{t}\right)\right)}\\ \;-\sum_{k=1}^{2}\frac{1}{2}\log\frac{\det\left(\mathrm{cov}\left({\hat{M}}^{k}\right)\right)}{\det\left(\mathrm{cov}\left({\hat{M}}_{\text{t}-\tau }^{k}|{\hat{M}}_{t}^{K}\right)\right)} \end{array}$$

with a normalization factor K:

$$\begin{array}{c} K\left(\left\{{\hat{M}}^{1},\;{\hat{M}}^{2}\right\}\right)=\frac{1}{2}\log\min_{k}\\ \;\left\{{\left(2\pi e\right)}^{\left|{\hat{M}}^{k}\right|}\det\left(\mathrm{cov}\left({\hat{M}}^{k}\right)\right)\right\} \end{array}$$

where ${\hat{M}}^{1}$ and ${\hat{M}}^{2}$ are bipartitions of $\hat{X}$; det(**C**) is the covariance of a matrix **C**, and cov($\hat{M}$) is the covariance of a system $\hat{M}$. The MIB for a given $\hat{X}$ is defined as:

$$\begin{array}{c} MIB\left(\hat{X}\right)={\left[{\hat{M}}^{1};\;{\hat{M}}^{2}\right]}_{\hat{X}}\;\text{for which}\frac{\text{EI}\left(\hat{X};\;\tau ,\left\{{\hat{M}}^{1},\;{\hat{M}}^{2}\right\}\right)}{K\left(\left\{{\hat{M}}^{1},\;{\hat{M}}^{2}\right\}\right)}\\ \;=\min\text{for all}\;{\hat{M}}^{1}\;\text{in}\;\hat{X} \end{array}$$

Then Φ Empirical is defined as the unnormalized EI over the MIB.

Φ Empirical can be calculated analytically for system $\hat{X}$. MATLAB code is available from http://www.ploscompbiol.org/article/fetchSingleRepresentation.action?uri = info:http://doi/10.1371/journal.pcbi.10011052.s001[16]. In our experiment, the noise, ${\hat{E}}_{t}$, was a Gaussian distribution with a mean of zero and a variance of one. *P* was set to one (for the generative equation) and thus the equation for activity of system $\hat{X}$ at time t is ${\hat{X}}_{t}=A_{1}{\hat{X}}_{t-1}+{\hat{E}}_{t}$ This is the same formula governing the 2003 Φ dynamics, $\hat{F}=\hat{F}\;CON\left(\hat{X}\right)+\hat{C}*\hat{R}$. Hence, the connection matrices from our paper, **C**, are represented as **A**_*1*_ in Φ Empirical notation (and **CON**($\hat{X}$) in 2003 Φ notation).

We calculated Φ(X) for τ = {1,2,3,4}. In addition we also performed an experiment using the value of $\sum_{\tau =1}^{4}\Phi (X)$.

## Competing interests

The authors declare that they have no competing interests.

## Authors’ contributions

MWH generated the SD measure, derived the relationships between SD and set theory, degree and the graph theory metrics, designed the experiments, generated graphs and analyzed data, and wrote and revised the manuscript. MFM was involved in the initial generation of variables to capture specialization and integration, designed the experiments, generated graphs, analyzed data and provided valuable feedback and revision on the manuscript. AW contributed some key observations, CWL contributed to the theoretical foundation and practical aspects of computation, ERR generated the idea behind the project, assisted in deriving the theoretical relationships between measures, designed the experiments, analyzed and interpreted data, and wrote and revised the manuscript. All authors read and approved the final manuscript.
